# Ten unique and charismatic new species of Microgastrinae wasps (Hymenoptera, Braconidae) from North America

**DOI:** 10.3897/zookeys.730.22869

**Published:** 2018-01-18

**Authors:** Jose Fernandez-Triana

**Affiliations:** 1 Canadian National Collection of Insects, Ottawa, Canada

**Keywords:** Microgastrinae, North America, conservation, species diversity, parasitoid wasps

## Abstract

Ten new species within four genera of Microgastrinae parasitoid wasps (Hymenoptera: Braconidae) are described from Canada and United States: *Diolcogaster ichiroi*, *Diolcogaster miamensis*, *Glyptapanteles pseudotsugae*, *Microgaster archboldensis*, *Microgaster syntopic*, *Microplitis altissimus*, *Microplitis jorgeluisi*, *Microplitis juanmanueli*, *Microplitis julioalbertoi*, and *Microplitis mariamargaritae*. The new taxa are significant because they represent the first North American records of a tropical group (species of the *basimacula* group in *Diolcogaster*), exemplify interesting ecological cases (niche-based host selection in *Glyptapanteles*, syntopic species in *Microgaster*), and showcase unique morphological features and/or altitudinal records (*Microplitis*). Most of the new species were collected in protected areas or areas with strong research programs (Archbold Biological Station and hammock forests near Miami, Florida; Great Sand Dunes National Park and Preserve, and Mount Evans Wilderness Area, Colorado; Sapelo Island, Georgia; Tonto National Forest, Arizona), and thus are also of value and interest for conservation and research efforts.

## Introduction

Microgastrine wasps are the second largest subfamily of Braconidae (Hymenoptera) with 2,700+ described species and an estimate 17,000-46,000+ worldwide (Rodriguez et al. 2013, Yu et al. 2016). It is also one of the most important groups in the biological control of agricultural and forestry pests worldwide (Whitfield 1997).

The Nearctic region (Canada, Greenland, and United States) has historically been considered as one of the best studied and best known natural regions of the planet. However, regarding microgastrine wasps that has not been the case: of the six major biogeographical regions considered in the 2016 version of Taxapad (Yu et al. 2016) the Nearctic is the second least diverse at species level, only surpassing the Australasian region. There are currently 330 described species of Microgastrinae in North America, and the progress has been relatively slow compared to other regions of the planet. After two seminal works from Muesebeck (1921, 1922), most of the new taxa recorded for the Nearctic have been described in papers dealing with single species, usually of interest in biological control (see references in Yu et al. 2016), with only few recent papers describing more than one species (e.g., Whitfield 1985, 2006, Williams 1981, 1988, Valerio et al. 2009, Fernandez-Triana 2010, Valerio and Whitfield 2015).

Hundreds of additional species for this region have been revealed by DNA barcoding (e.g., Smith et al. 2013), but the west coast and southernmost areas of North America, which also happen to be the most diverse, have barely been analyzed, suggesting that the actual species diversity in the region will be several times higher –when more studies are done.

To highlight how few we currently know about the group in the region, we describe below ten new species within four genera of Microgastrinae. All of the new species represent significant and in many cases unique records, as this paper intends to bring further attention to special conservation areas in North America.

## Methods

All specimens studied for this paper are deposited in the Canadian National Collection of Insects, Ottawa (CNC).

Morphological terms and measurements of structures follow those used by Mason (1981), Huber and Sharkey (1993), Whitfield (1997), Karlsson and Ronquist (2012), and Fernandez-Triana et al. (2014).

The abbreviations T1, T2, and T3 refer to metasomal mediotergites 1, 2, 3; F2/3/14/15/16 refer to length of antennal flagellomeres 2, 3, 14, 15 and 16; and L and W refer to length and width respectively. The description of the new species contains some ratios commonly used in taxonomic studies of Microgastrinae, but raw measurements of morphological structures (in mm) are also provided as they allow for additional ratios to be explored in the future, if needed. When presenting the raw measurements, the holotype value is given first, followed by the range of other specimens between parentheses.

For some specimens DNA barcodes (the 5’ region of the cytochrome c oxidase I (CO1) gene, Hebert et al. 2003) were available. DNA extracts were obtained from single legs using a glass fibre protocol (Ivanova et al. 2006). Total genomic DNA was re-suspended in 30 μl of dH2O, a 658-bp region near the 5’ terminus of the CO1 gene was amplified using standard primers (LepF1–LepR1) following established protocols (http://v4.boldsystems.org/index.php), and a composite sequence was generated for all successful amplifications. All information for the sequences associated with each individual specimen can be retrieved from the Barcode of Life Data System (BOLD) (Ratnasingham and Hebert 2007).

Photos were taken with a Keyence VHX-1000 Digital Microscope, using a lens with a range of 10–130 ×. Multiple images were taken of a structure through the focal plane and then combined to produce a single in-focus image using the software associated with the Keyence System. Plates were prepared using Microsoft PowerPoint 2010.

A map with the distribution of the species was generated using SimpleMappr (Shorthouse 2010).

For states of the United States and for Canadian provinces/territories, acronyms consisting of two capital letters are used, following Canada Post (http://www.canadapost.ca/tools/pg/manual/PGaddress-e.asp).

## Results

Ten new Nearctic species of Microgastrinae are described below, arranged alphabetically by genus (and species within every genus). Every new species is compared and diagnosed against all other previously described Nearctic species of that genus. The new taxa are significant because they represent the first North American records of a tropical group (species of the *basimacula* group in *Diolcogaster*), exemplify interesting ecological cases (niche-based host selection in *Glyptapanteles*, syntopic species in *Microgaster*), and showcase unique morphological features and/or altitudinal records (*Microplitis*).

Four of the described species were found in south Florida, three were found in the mountains of Colorado, and another two species were distributed across the west coast of North America (Figure 11). In most cases, the new species were collected or reared in protected areas and/or areas with strong research programs (Archbold Biological Station and hammock forests near Miami, FL; Great Sand Dunes National Park and Preserve, and Mount Evans Wilderness Area, CO; Sapelo Island, GA; Tonto National Forest, AZ), and thus by describing them it is hoped further attention is brought into their conservation.

### Genus *Diolcogaster* Ashmead, 1900

There are nine described species of *Diolcogaster* in the Nearctic (Yu et al. 2016), but many more await description. The two new species described below are very distinct from any previously described species in North America, as they both belong to the *basimacula* species-group, a mostly a tropical group, with dozens of undescribed species worldwide. The finding of these species in mainland North America is unique, but not entirely surprising as south Florida has close biogeographical affinities with the Neotropical fauna (e.g., Snyder et al. 1990). The description of these two new species will hopefully bring further attention to the unique values of the biodiversity in south Florida and the need to preserve those ecosystems.

#### Diolcogaster ichiroi

Taxon classificationAnimaliaHymenopteraBraconidae

Fernandez-Triana sp. n.

http://zoobank.org/717E90BC-7742-4D0D-80B9-9A4FD5998D82

Fig. 1

##### Holotype.

Female, CNC, UNITED STATES. Holotype locality: Archbold Biological Station, Highlands County, Florida, USA.

##### Holotype labels.

First label: USA: FL, Highlands Co./Archbold Biol. Sta./1–8.vi.1987/Dr.X.Wahl. Second label: CNC483614.

##### Paratypes.

2♀, 5 ♂ (CNC) from the same locality than holotype. Voucher codes: CNC483650–CNC483652, CNC489768, CNC489820, CNC489849, CNC526748. Collecting dates: 1-22.vii.1987 and 18.iii-4.iv.1988, some specimens collected with a Malaise trap.

**Figures 1.**
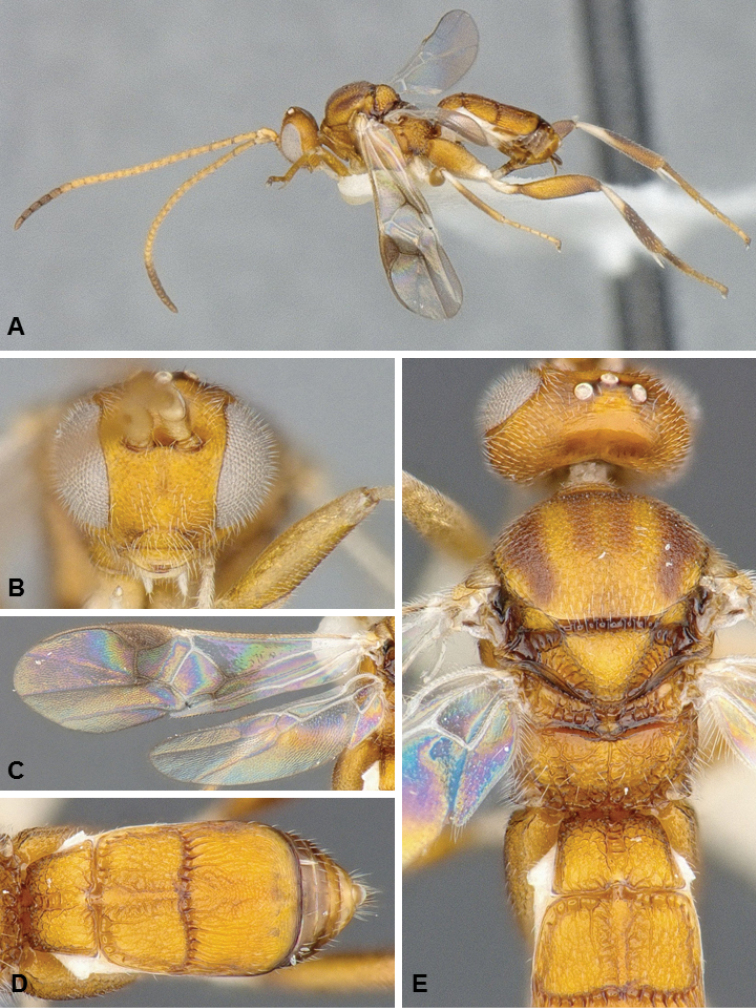
*Diolcogaster ichiroi*, female holotype.

##### Diagnosis.

**Figures 2.**
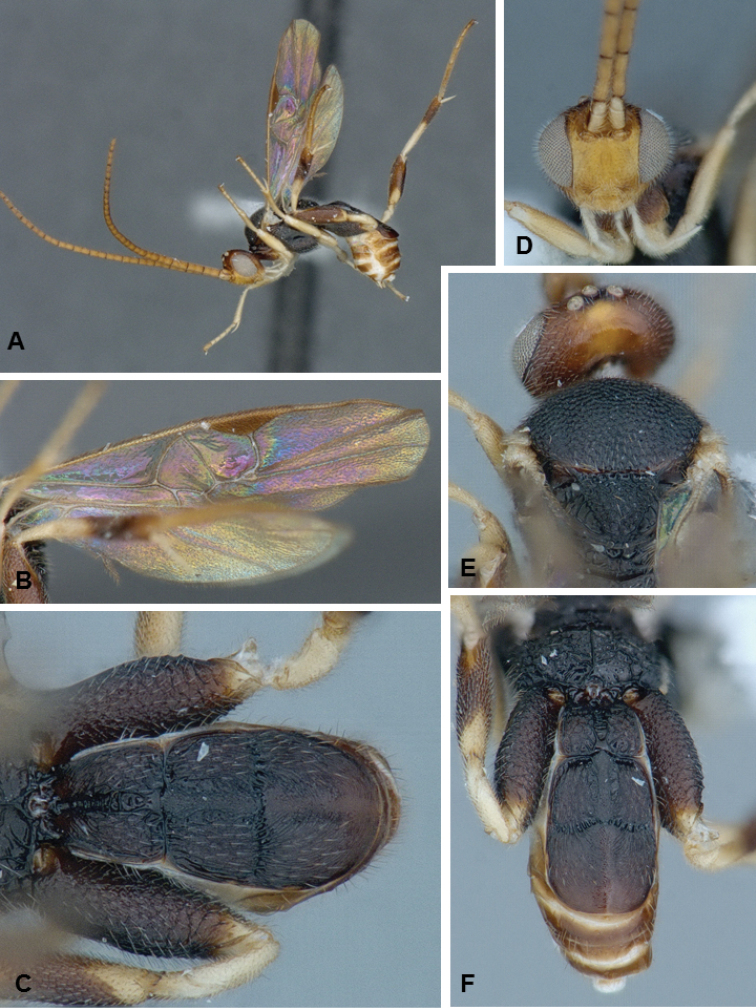
*Diolcogaster miamensis*, male paratype.

*Diolcogaster ichiroi* and *D. miamensis* (see next species below) are very distinct and unique among all known species of *Diolcogaster* from North America, based on its tergites 1–3 forming a carapace that covers most of the metasoma. That is the main distinguishing feature of the *basimacula* group, which is very speciose in the Old World tropics but until now had never been reported from the New World (although numerous undescribed species from the Neotropics are found in collections). *Diolcogaster ichiroi* (body mostly yellow, with some small brown areas; fore wing centrally with some veins transparent) has different coloration than *D. miamensis* (head yellow frontally, orange in the back; meso- and metasoma mostly black; fore wing centrally with veins brown); the two species also differ in the shape and sculpture of T2 (anterior and posterior margin of T2 more or less straight in *ichiroi*, curved in *miamensis*, compare Figs 1D, 2F), as well as setae thickness near apex of ovipositor sheaths (all setae of same thickness in *miamensis*, a couple of setae thicker than the rest in *ichiroi*).

##### Description.

Female. Body color mostly yellow (with some brown spots on metasoma; T4+ dark brown; anterior laterotergies and sternites, pro- and mesocoxae, all trochanters and trochantellus, anterior 0.2–0.3 of tibiae, and metatibial spurs white; antenna flagellomeres mostly yellow, but with tip brown. Wings mostly hyaline but with a couple of infumate spots, some veins brown and some transparent, pterostigma brown. Body mostly coarsely sculptured. Scutoscutellar sulcus with 9–10 costulae. Hind wing with vannal lobe straight to slightly concave and centrally without setae. Tarsal claws simple. T1–3 forming a carapace that covers most of metasoma, T4+ scarsely visible. Ovipositor sheaths relatively short, with long setae, including a couple of thicker setae near apex of sheaths. **Body measurements (mm).** Body L: 2.3 (2.0–2.1); fore wing L: 2.1 (1.8–2.0); ovipositor sheaths L (approximate measurement): 0.12 (0.11); metafemur L/W: 0.65/0.18 (0.65/0.18); metatibia L: 0.81 (0.81); metatibia inner/outer spurs L: 0.21/0.16 (0.21/0.15); first segment of metatarsus L: 0.38 (0.37); F2/3/14/15/16: 0.19/0.17/0.09/0.09/0.11 (0.20/0.17/0.09/0.09/0.11); ocular–ocellar line: 0.10 (0.10); interocellar distance: 0.10 (0.11); posterior ocellus diameter: 0.06 (0.07).

Male. As female, but darker (more extensive brown areas on anteromesoscutum, mesoscutellar-axillar complex, metascutellum and metasomal terga).

##### Distribution.

United States: FL. Only known from the type locality (Archbold Biological Station).

##### Etymology.

This unique and remarkable species is named to honor the truly unique and remarkable Ichiro Suzuki, my favorite baseball player and one the best ever to play the game. At the time the research for this paper was being conducted, Ichiro was still playing for a Florida team and thus naming a species endemic from Florida after him made complete sense. Unfortunately, the new owners of the Miami Marlins did not keep Ichiro, an unpopular decision not liked by many Marlins fans. Hopefully soon another Major League team gives the Universal Hit King the chance to continue his extraordinary career in baseball.

##### Notes.

Both this species and the next one are examples of mostly tropical groups that in North America are only found in south Florida (e.g., Snyder et al. 1990). Altogether with other microgastrine wasps recently described from that area (see Fernandez-Triana and Boudreault 2016, as well as the two new *Microgaster* species being described below in this paper), all of these taxa highlight the importance of biodiversity studies in south Florida and the need to increase conservation efforts there.

#### Diolcogaster miamensis

Taxon classificationAnimaliaHymenopteraBraconidae

Fernandez-Triana sp. n.

http://zoobank.org/85DAD587-5462-46E7-A8AE-1138554AE4F0

Fig. 2

##### Holotype.

Female, CNC, UNITED STATES. Holotype locality: Hammock forest on Chekika State Park Recreation Area, SW of Miami, Dade County, Florida, USA.

##### Holotype labels.

First label: FLA: Dade Co; Chekika St./Rec. Area, 50 km SW Miami/1.v–2.viii.1985, S&J Peck/Grossman Hammock For./malaise-FIT. Second label: CNC735735.

##### Paratypes.

1 ♂ (CNC) from Archbold Biological Station, Highlands County, Florida, USA. Voucher code: CNC489838. Collecting dates: 18–22.iii.1987.

##### Diagnosis.

See *Diolcogaster ichiroi* above for details on how these two species are distinct from each other and from all other known *Diolcogaster* in North America.

##### Description.

Female. Body coloration varied: head yellow frontally, orange in the back, antenna flagellomeres mostly yellow, but with tip brown; meso- and metasoma mostly black, with some small areas light brown; legs mostly yellow-white, with most of metafemur and posterior 0.5 of metatibia brown, and metatarsus yellow-brown. Wings mostly hyaline, with slightly infumate spot below pterostigma, most veins brown and pterostigma brown. Body mostly coarsely sculptured. Scutoscutellar sulcus with 8 costulae. Hind wing with vannal lobe straight to slightly concave and centrally without setae. Tarsal claws simple. T1–3 forming a carapace that covers rest of metasoma. Ovipositor sheaths relatively short, with long setae (but all of same thickness). **Body measurements (mm).** Body L: 2.2; fore wing L: 2.2; ovipositor sheaths L (approximate measurement): 0.15; metafemur L/W: 0.68/0.22; metatibia L: 0.87; metatibia inner/outer spurs L: 0.27/0.20; first segment of metatarsus L: 0.41; F2/3/14/15/16: 0.23/0.21/0.12/0.11/0.14; ocular–ocellar line: 0.09; interocellar distance: 0.08; posterior ocellus diameter: 0.07.

Male. As female.

##### Distribution.

United States: FL. Only known from two localities in south Florida.

##### Etymology.

Named after the Miami metropolitan area (also known as Greater Miami or South Florida), where the holotype locality is found, to highlight the great natural values of the area and to bring further attention to the conservation and appreciation of nature in south Florida.

##### Notes.

See Notes above (under *Diolcogaster ichiroi*) for more details on the conservation value of these species. Both specimens of *D. miamensis* were collected with Malaise traps.

### Genus *Glyptapanteles* Ashmead, 1904

There are 18 described species of *Glyptapanteles* in the Nearctic (Yu et al. 2016), but many more await description. The new species described below is very distinctive because on its enlarged eyes, the first North American species of the genus with that character. A related genus, *Distatrix*, shares this feature, but the new species clearly belongs to *Glyptapanteles* due to the presence of two pronotal furrows (*Distatrix* only has one pronotal furrow, see Mason 1981) and the host families it parasitizes. From a biological perspective, the new species is also unique, as it parasitizes different host caterpillars feeding on Douglas fir across a range of 2,500 km in western North America.

#### Glyptapanteles pseudotsugae

Taxon classificationAnimaliaHymenopteraBraconidae

Fernandez-Triana sp. n.

http://zoobank.org/58D06EA4-35DE-4D8B-91D1-E2F897D8EA87

Fig. 3

##### Holotype.

Female, CNC, UNITED STATES. Holotype locality: Aztec Peak, Arizona, USA.

##### Holotype labels.

First label: Aztek Pk., AR./coll. vi-1-77/em. vi-24/Torg. 1977 7065A. Second label: Ex *Orgya pseudotsugata*. Third label: Hopk. US/65254. Fourth label: CNC666525.

##### Paratypes.

11♀, 17 ♂ (CNC) from the following localities. Canada: AB, Pincher Creek; BC, Carquile; BC, Elko; BC, Mount Lolo; BC, Nelson; BC, Lake Williams. United States: AZ, Aztec Peak, Tonto National Forest; CA, El Dorado County, Iron Mountain; CA, San Bernardino County, Sky Forest; CA, Stowe Reservoir; CA, Modoc County, Tom’s Creek; OR, Chiloquin Ridge; OR, Forth Klamath. Voucher codes: CNC841809– CNC841836. All of the specimens were reared, with emergence dates from early June to early August.

**Figures 3.**
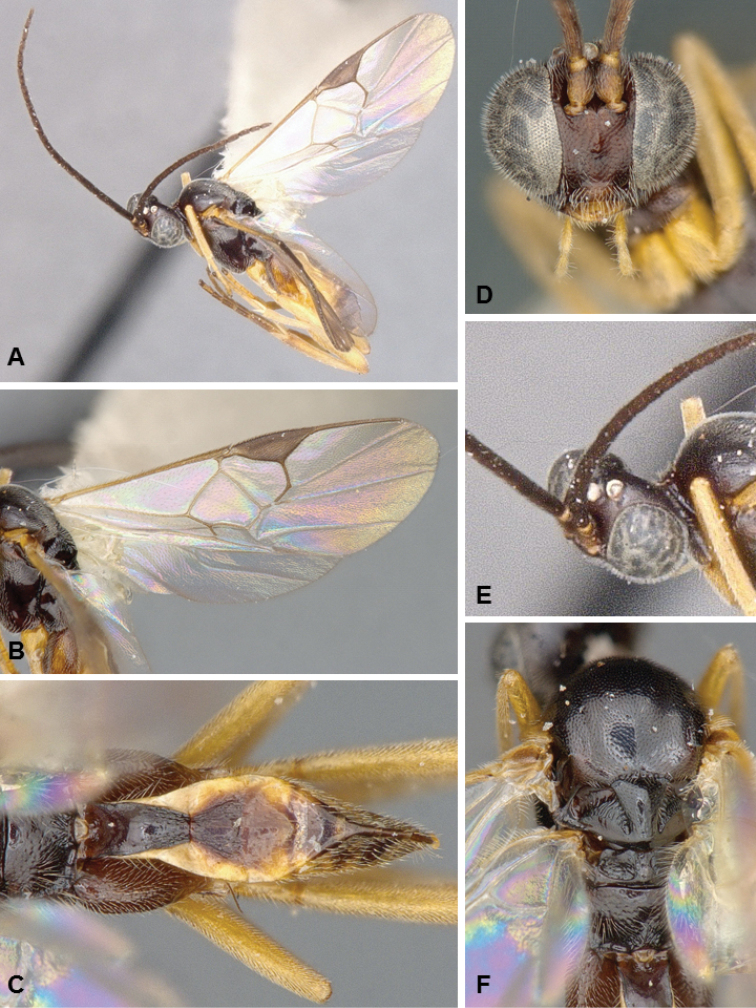
*Glyptapanteles pseudotsugae*, female holotype.

##### Diagnosis.

The enlarged eyes and ocelli of *G. pseudotsugae* are unlike those of any other described species of *Glyptapanteles* in North America –all of which have normal-sized eyes. The antenna of females is also rather long, with the last flagellomeres not significantly reduced, as it is the case with most Microgastrinae female specimens. The size of eyes and ocelli, the relatively long antenna, and the yellow-brown body coloration are all morphological features that strongly suggest this species is nocturnal or crepuscular – see Quicke (2015) for a summary and further references on the suite of characters that are typical of nocturnal/crepuscular parasitoid wasps. The caterpillar hosts are also unique among known hosts of Microgastrinae (see below).

##### Description.

Female. Body mostly brown to dark brown (except for yellow scape, pedicel, labrum, mandibles, palpi, tegula; humeral complex half yellow and half brown; T3+ partially yellow; anterior laterotergites and sternites mostly yellow; hypopygium sometimes partially yellow); most of legs yellow, but metacoxa, apical 0.1 of metafemur and metatibia, and metatarsus brown. Wings hyaline, pterostigma brown, veins mostly transparent (except for a few veins closer to pterostigma). Body mostly smooth and shiny, at most with fine, shallow and sparse punctures; propodeum with small striae around nucha; apical 0.3 of T1 and most of T2 (except centrally) with relatively coarse longitudinal striation. Head with eyes and ocelli enlarged. Protarsus with a thick and curved seta. Fore wing with veins r and 2RS meeting at a sharp angle, with vein 3RSa being a very small stub; vein R1 longer than pterostigma. Legs with tarsal claws simple. T1 narrowing towards posterior margin, T2 subtriangular (trapezoidal). Ovipositor sheaths with a few, large setae near tip. **Body measurements (mm).** Body L: 3.3 (3.2–3.7); fore wing L: 3.6 (3.7–4.1); ovipositor sheaths L: 0.15–0.20 (approximate measurement); metafemur L/W: 1.02/0.25 (1.04/0.25); metatibia L: 1.18 (1.22–1.24); metatibia inner/outer spurs L: 0.33/0.26 (0.32–0.36/0.24–0.26); first segment of metatarsus L: 0.48 (0.50–0.55); F2/3/14/15/16: 0.32/0.30/0.15/0.14/0.16 (0.31–0.32/0.29–0.30/0.14–0.15/0.13/0.15–0.16); ocular–ocellar line: 0.06 (0.04–0.07); interocellar distance: 0.12 (0.10–0.13); posterior ocellus diameter: 0.11 (0.11–0.12).

Male. As female, but eyes not enlarged, and general coloration, especially on metasoma, darker.

##### Variation.

Compared to the US specimens, the Canadian specimens are darker (dark brown to black scape, clypeus, labrum and most tergites) and also slightly larger (0.1–0.2 mm longer wings and body).

##### Distribution.

Western North America, from 33°–52°N. Canada: AB, BC. United States: AZ, CA, OR.

##### Host data.

The US specimens of *Glyptapanteles pseudotsugae* were all reared from the Douglas-fir tussock moth, *Orgya pseudotsugata* (McDunnough, 1921) (Lymantriidae), while the Canadian specimens were reared from three different species of Geometridae: the Spruce-fir looper *Macaria signaria dispuncta* (Walker, 1860), the Brown-lined looper *Neoalcis californiaria* (Packard, 1871), and *Pero behrensarius behrensarius* (Packard, 1871). Most of the specimens we examined had remnants of the host larva and/or the wasp cocoon preserved (kept in a gel capsule, pinned or glued to the paper where the adult wasp was mounted); based on that evidence, the parasitoid is considered to be solitary. *Glyptapanteles pseudotsugae* is the first species of Microgastrinae recorded attacking those four species of Lepidoptera. [There actually is an earlier mention of this wasp species, as an unidentified “*Apanteles* sp.”, in a previous publication studying the parasitoids and predators of *Orgya pseudotsugata* (Dahlsten et al. 1977); that is to be expected as all *Glyptapanteles* species were considered to belong to *Apanteles* until Mason (1981) split the latter genus into several]. The four lepidopteran hosts recorded above all feed on Douglas fir *Pseudotsuga menziesii* (Mirb.) Franco.

##### Etymology.

Named after the genus name of the Douglas fir, *Pseudotsuga*, as that plant harbours all caterpillar species that are host of the parasitoid wasp in North America.

##### Notes.

*Glyptapanteles pseudotsugae* is an example of niche-based selection of caterpillar hosts by a parasitoid wasp, as all of the Lepidoptera species recorded here coexist on fir forests in North America (e.g., Mason 1987). That contrasts with the recorded information for most Microgastrinae wasps, which usually parasitize taxonomically related hosts. Despite the relatively wide geographical distribution of the species in western North America (the distance between the southernmost known specimens in central Arizona and the northernmost known specimens in southern British Columbia is approximately 2,500 km), and the different hosts species parasitized across the wasp range, only minor morphological differences are apparent, and thus the US and Canadian wasp specimens are here considered to be conspecific. Many of the US specimens from the type series detailed above come from Dahlsten et al. (1977), although those authors saw additional specimens not seen nor studied for this paper. No molecular data is known for this species.

### Genus *Microgaster* Latreille, 1804

There are 18 described species of *Microgaster* in the Nearctic (Yu et al. 2016), with many more undescribed. The two new species described below are very distinctive because of their large body size and characteristic color patterns, as well as the arrangement of placodes on the antennal flagellomeres, unique among all other known species of the genus in North America. Based on the strong morphological similarities and the shared geographic distribution (at least partially), they very likely represent an example of sympatric speciation. Furthermore, both new species were mostly found in important conservation areas of south Florida and Georgia; its description also intends to increase public awareness of the biodiversity values of those areas.

#### Microgaster archboldensis

Taxon classificationAnimaliaHymenopteraBraconidae

Fernandez-Triana sp. n.

http://zoobank.org/C115F955-C73A-4B06-8EB1-BA0292977DAD

Fig. 4

##### Holotype.

Female, CNC, UNITED STATES. Holotype locality: Archbold Biological Station, Highlands County, Florida, USA.

##### Holotype labels.

First label: U.S.A. FL: Highlands Co./Archbold Biol. Sta./1-8.vi.1987, D. B. Wahl/CNC489773.

##### Paratypes.

1♀, 8 ♂ (CNC) from the same locality than holotype. Voucher codes: CNCHYM 01662, CNCHYM 01663, CNCHYM 01665, CNC483424, CNC489814, CNC654633–CNC654636. Collecting dates: 1–29.vi.1987 and 18–24.viii.1987, some specimens collected with Malaise trap and others with flight interception traps.

**Figures 4.**
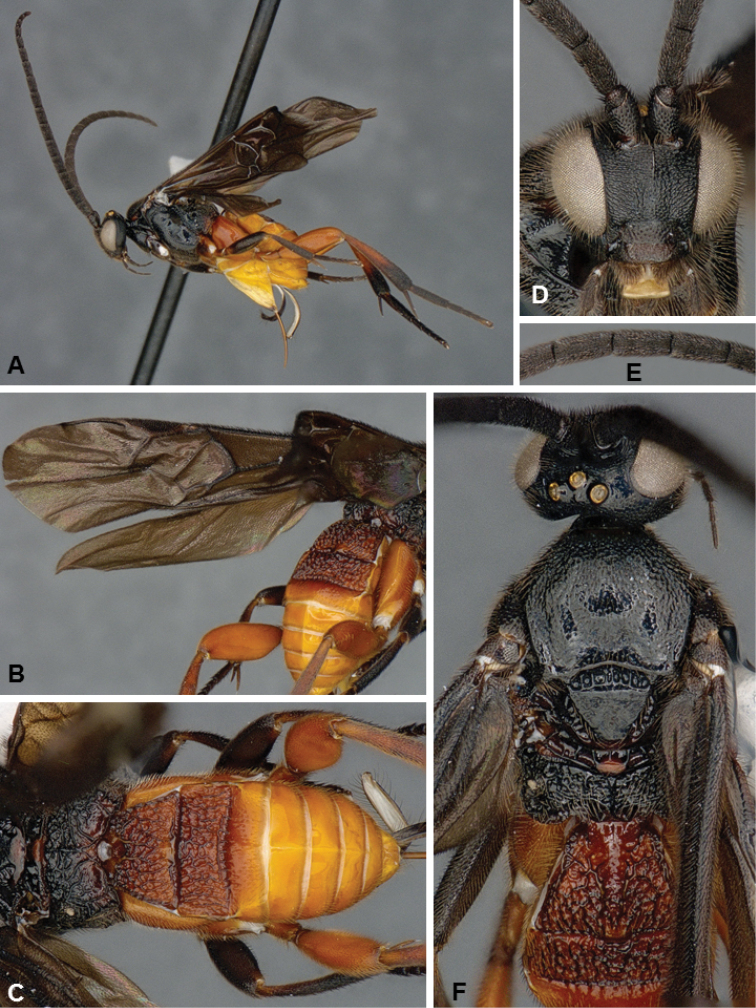
*Microgaster archboldensis*, female holotype.

##### Diagnosis.

**Figures 5.**
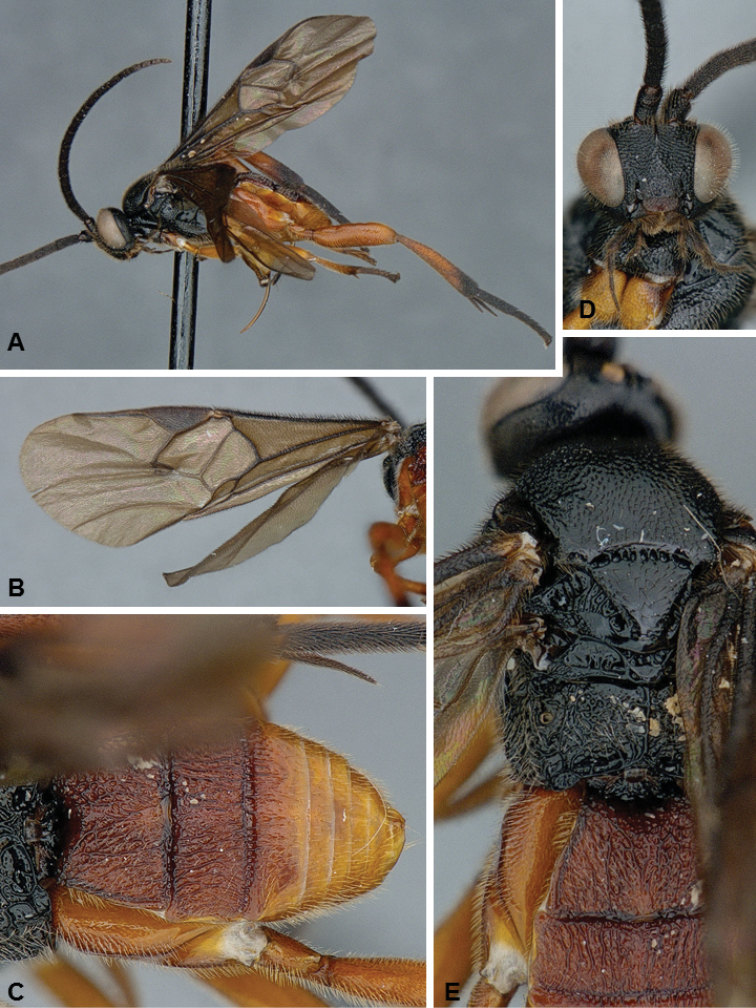
*Microgaster syntopic*, female holotype.

*Microgaster archboldensis* and *M. syntopic* are very distinct and unique among all known species of *Microgaster* from North America because of its color pattern, body size, and flagellomeres with three rows of placodes. The latter is the most important feature, as it had not been recorded from any known Nearctic species until now, and it was rather considered to characterize the different but related genus *Hygroplitis* (e.g., Mason 1981). However, *M. archboldensis* and *M. syntopic* clearly belong to *Microgaster* as they both have pectinated tarsal claws, pleated hypopygium, apical tarsomeres not enlarged, and body not partially depressed (whereas *Hygroplitis* has simple tarsal claws, inflexible hypopygium, apical tarsomeres enlarged and body partially depressed). Besides morphology, available DNA barcodes clearly place these new species within *Microgaster* and not *Hygroplitis*. *Microgaster archboldensis* can in turn be separated from *M. syntopic* because of different color of front and mid legs, part of propodeum, scutellar disc and metanotum (compare Figs 4A, C, F versus Figs 5A, C, E); longer ovipositor sheaths, body length and fore wing length; and some additional minor differences in mesopleuron sculpture, and number of costulae in scutoscutellar sulcus. From a molecular perspective (DNA barcoding), the two species differ in 27 base pairs (bp), which amounts to a rather significant difference of more than 4.5% bp (the available sequences for *M. archboldensis* represent almost complete barcodes with 626–627 bp, but the available sequences for *M. syntopic* are shorter at only 422–593 bp).

##### Description.

Female. Head and mesosoma mostly black (except for reddish-orange spots on posterior half of propodeum, posterior margin of scutellar disc and central part of metanotum); metasoma with T1–3 reddish-orange, T4+ orange-yellow, hypopygium mostly yellow to yellow-white; front legs entirely dark brown to black; mid legs almost entirely dark brown to black (except for coxa, trochanter and trochantellus, which are partially orange and partially dark brown); hind legs mostly orange (except for posterior 0.3 of metatibia, metatibial spurs and metatarsus which are dark brown to black); wings strongly infumated, pterostigma and veins dark brown to black. Flagellomere with three rows of placodes. Scutoscutellar sulcus with 5–6 costulae. Hypopygium pleated. Tarsal claws pectinate. **Body measurements (mm).** Body L: 5.2 (5.4); fore wing L: 5.3 (5.5); ovipositor sheaths L: 1.28 (1.30); metafemur L/W: 1.64/0.51 (1.70/0.54); metatibia L: 1.96 (2.02); metatibia inner/outer spurs L: 0.76/0.47 (0.76/0.51); first metatarsus segment L: 0.97 (0.96); F1/2/3/14/15/16: 0.40/0.41/0.42/0.21/0.18/0.20 (0.40/0.43/0.43/0.24/0.20/0.21).

Male. As female.

##### Molecular data.

Three barcode-compliant sequences, representing BIN BOLD:AAZ7880 in BOLD.

##### Distribution.

United States: FL. Only known from the type locality, Archbold Biological Station.

##### Etymology.

Named after the Archbold Biological Station in Florida, US, to recognize the extraordinary fauna of Microgastrinae (and certainly of many other taxa) that it harbors and protects.

##### Notes.

In spite of the relatively strong morphological and molecular differences, *Microgaster archboldensis* still seems very close to *M. syntopic*, and both are at least partially sympatric in central Florida. See Notes above (under *Diolcogaster ichiroi*) for more details on the conservation value of all these species.

#### Microgaster syntopic

Taxon classificationAnimaliaHymenopteraBraconidae

Fernandez-Triana sp. n.

http://zoobank.org/8F6554C3-FEE9-40D9-ADC7-414E4A9C1990

Fig. 5

##### Holotype.

Female, CNC, UNITED STATES. Holotype locality: Archbold Biological Station, Highlands County, Florida, USA.

##### Holotype labels.

First label: USA: FL, Highland Co./ Lake Placid/ Archibold Biol. Sta./ 8-14.ix.1987; FIT/ BRC HYMN TEAM. Second label: CNC483215.

##### Paratypes.

2♀, 5 ♂ (CNC) from the same locality than holotype, collecting dates: 26.iv.1967, 23.v.1967, 1–8.vi.1987, 9–22.vi.1987, 21.ix.1987; 3♀, 2 ♂ (CNC) from USA, FL, Alachua County, Gainesville, American Entomological Institute, collecting dates: 1–15.ix.1987, 29.ix.1986, 6.x.1986, 24.vi–13.viii.1987; 4 ♂ (CNC) USA, GA, McIntosh County, Sapelo Island, Oak forest, collecting dates: 20.vi–18.vii.1987, 15.vii-9.ix.1987. Voucher codes: CNCHYM 01664, CNCHYM 07428, CNCHYM 07429, CNC280981, CNC280993, CNC280996, CNC483414, CNC483415, CNC483419, CNC483355, CNC489769, CNC489772, CNC489778, CNC841837–CNC841839. Some specimens were collected with Malaise trap and others with flight interception traps.

##### Diagnosis.

See *Microgaster archboldensis* above for details on how these two species are distinct from each other and from all other known *Microgaster* in North America.

##### Description.

Female. Head and mesosoma black; metasoma with T1–3 reddish-orange, T4+ and hypopygium orange-yellow; all legs mostly reddish-orange (except for posterior 0.2–0.1 of metatibia, metatibial spurs and metatarsus which are dark brown to black); wings strongly infumated, pterostigma and veins dark brown to black. Flagellomere with three rows of placodes. Scutoscutellar sulcus usually with 7–8 costulae (rarely with 5–6). Hypopygium pleated. Tarsal claws pectinate. **Body measurements (mm).** Body L: 4.6 (4.6–5.2); fore wing L: 4.9 (4.8–5.3); ovipositor sheaths L: 0.85 (0.86–0.94); metafemur L/W: 1.56/0.46 (1.50–1.63/0.50–0.54); metatibia L: 1.82 (1.82–1.98); metatibia inner/outer spurs L: 0.66/0.41 (0.70–0.78/0.46–0.50); first metatarsus segment L: 0.83 (0.80–0.98); F1/2/3/14/15/16: 0.40/0.40/0.40/0.19/0.18/0.20 (0.38–0.43/0.39–0.45/0.40–0.44/0.19–0.20/0.16/0.19–0.20).

Male. As female.

##### Molecular data.

Two sequences, one of them barcode-compliant, representing BIN BOLD:AAZ7881 in BOLD.

##### Distribution.

United States: FL, GA. Only known from two localities in Florida and one in Georgia.

##### Etymology.

Derived from Greek, ‘syntopic’ meaning ‘from the same place’, a term used in Zoology to reference two or more related species which can occupy the same locality/habitat, and could possibly hybridize or even be sister species (see explanation of the concept in Rivas 1964). The name refers to this species being syntopic with *Microgaster archboldensis* (at least around Archbold Biological Station, where both species were collected, sometimes on the same date and by the same Malaise trap).

##### Notes.

See Notes above (under *Diolcogaster ichiroi*) for more details on the conservation value of these species.

### Genus *Microplitis* Foerster, 1863

There are 36 described species of *Microplitis* in the Nearctic (Yu et al. 2016), but many more remain undescribed in the region (e.g., Fernandez-Triana 2010). The five new species described below are very distinctive on different accounts (relatively large or small body size, extremely long metasoma, unique hypopygium and/or ovipositor sheath shapes, wing coloration, elongated mouth parts). One of the new species represents the highest altitude record of a microgastrine wasp ever reported in North America (and indeed one of the highest ever recorded for that group in the world). Another new species has the longest metasoma ever observed in the Microgastrinae subfamily. In all cases, most of the specimens were collected in protected or significant areas. Their description intend to bring further appreciation of the extraordinary diversity and uniqueness of parasitoid wasps.

#### Microplitis altissimus

Taxon classificationAnimaliaHymenopteraBraconidae

Fernandez-Triana sp. n.

http://zoobank.org/21D8B7CF-8BBE-4156-90B7-67F1C854AD8E

Fig. 6

##### Holotype.

Female, CNC, UNITED STATES. Holotype locality: Mount Evans, 3,658m, Clear Creek County, Colorado, USA.

##### Holotype labels.

First label: Mt. Evans, COLO./12,000’ 3 Aug./W.R.W.Mason ‘61. Second label: MIC CNC666529.

##### Paratype.

1 ♀, 2 ♂ (CNC). Same locality than holotype, but collected at altitudes ranging from 4,023m (female) to 4,267m (males); collecting dates from 25.vii–4.viii.1961.

**Figures 6.**
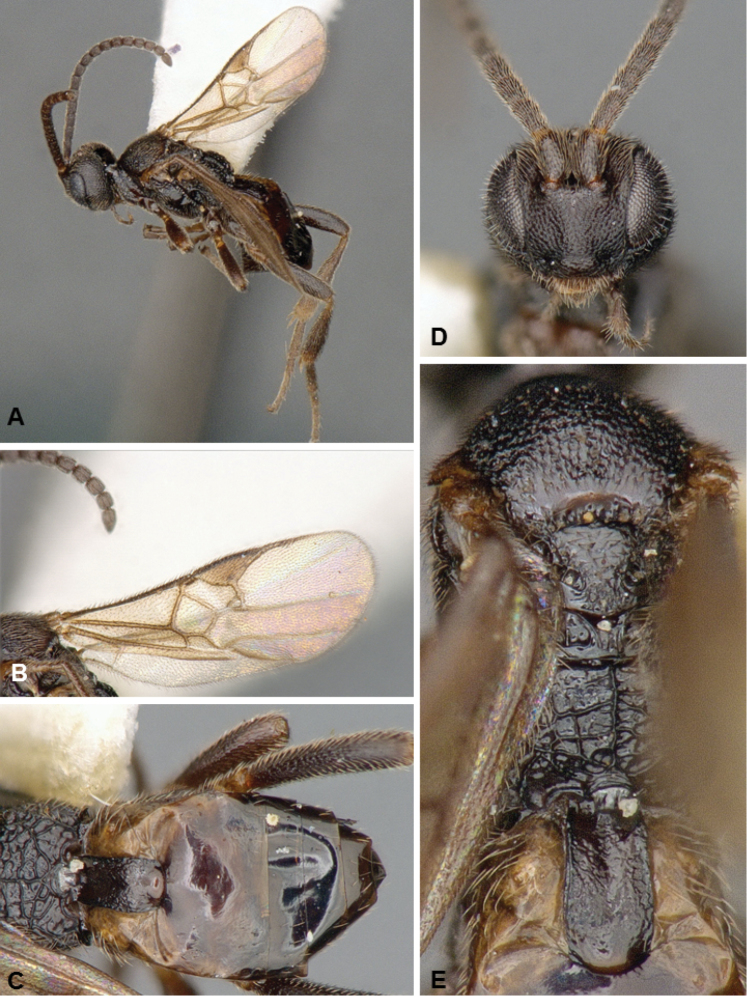
*Microplitis altissimus*, female holotype.

##### Diagnosis.

This species can be separated from all other described species of *Microplitis* in North America by the combination of the following features: small size (body length 2.0–2.1 mm, fore wing length 1.7–1.8 mm), entirely dark brown to black coloration (including all legs), slightly infumated wings, very short antenna (its length not surpassing the length of mesosoma), apical flagellomeres cubic (about as long as wide), and high altitudinal distribution.

##### Description.

Body dark brown to black (except for metatibia and al tarsi light brown). Wings slightly infumated, veins and pterostigma mostly brown. Head and mesosoma (dorsally) finely sculptured, metasoma mostly smooth dorsally, except for finely sculptured T1. Ovipositor sheaths smooth, almost without setae. Head not elongate; malar line shorter than mandible base; labrum, mandibles and glossa not enlarged; antennal flagellomeres 14–15 cubic (about as long as wide). Hypopygium not elongate and not projecting beyond last tergum. Ovipositor sheaths relatively very small, barely visible beyond hypopygium. Fore wing with vein R1 much shorter than pterostigma. Legs with tarsal claws simple. **Body measurements (mm).** Body L: 2.1 (2.0); fore wing L: 1.8 (1.7); ovipositor sheaths L: 0.15 (approximate measurement); metafemur L/W: 0.59/0.15; metatibia L: 0.77; metatibia inner/outer spurs L: 0.13/0.12; first segment of metatarsus L: 0.33; F1/2/3/14/15/16: 0.14/0.11/0.10/0.07/0.07/0.11; ocular–ocellar line: 0.13; interocellar distance: 0.09; posterior ocellus diameter: 0.04.

Males. As females but with antenna of more normal length.

##### Distribution.

United States: CO.

##### Etymology.

From the Latin adjective ‘altissimus’, meaning ‘the highest’, referring to the locality at which all specimens were collected, currently the highest altitude of any known species of Microgastrinae in North America.

##### Comments.

No biological or molecular data is known for this species. The small body and wings size, reduced antenna and short flagellomeres, and the dark coloration are all adaptions to living in a very cold, windy and harsh environment such as Mount Evans. The two male speciens were collected at 4,267m, by far the highest altitude recorded for any Microgastrinae in North America.

#### Microplitis jorgeluisi

Taxon classificationAnimaliaHymenopteraBraconidae

Fernandez-Triana sp. n.

http://zoobank.org/AF9B3584-E65F-43AF-A93D-2B9162A2D0A7

Fig. 7

##### Holotype.

Female, CNC, UNITED STATES. Holotype locality: Camp Maxey, Lamar County, Texas, USA.

##### Holotype labels.

First label: USA: TX, Lamar Co./Camp Maxey/21.IX.-21.X.2003, MT/W. Godwin, SFASU/grassy site, lot # 88. Second label: MIC 000683.

**Figures 7.**
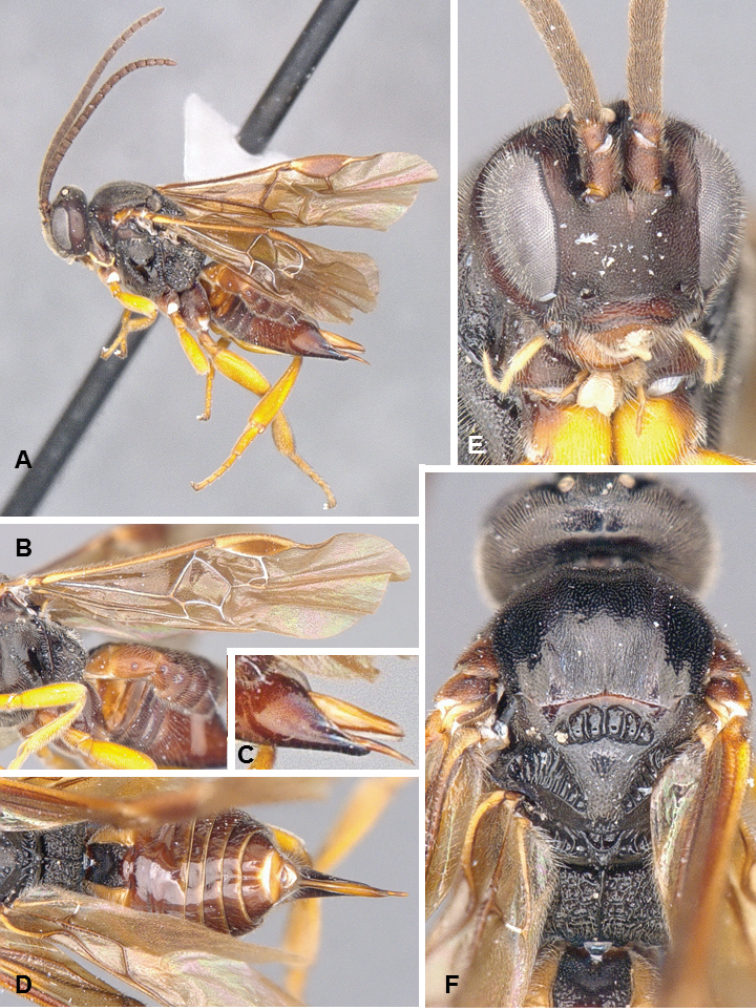
*Microplitis jorgeluisi*, female holotype.

##### Diagnosis.

The combination of enlarged and acute hypopygium; long, thick, and smooth ovipositor sheaths; bilobate glossa; metafemur relatively very short and robust; and head laterally with antennal socket shelf-like are very distinctive and unique among all described species of Nearctic *Microplitis*. See above (under *M. julioalbertoi*) for further diagnostic characters to separate both species.

##### Description.

Head and mesosoma black (except for orange-brown clypeus and labrum), metasoma mostly reddish-brown, legs mostly orange-yellow (except for brown coxae). Wings entirely infumated, veins dark brown, pterostigma brown with yellow spot at base. Head finely sculptured, mesosoma and metasoma mostly smooth dorsally (except for finely sculptured T1). Ovipositor sheaths smooth, almost without setae (only very few and short setae near apex). Head not elongate; malar line shorter than mandible base; labrum and mandibles not enlarged; glossa slightly elongate and bilobate. Hypopygium elongate and sharply acute, projecting considerably beyond last tergum. Ovipositor sheaths relatively long. Fore wing with vein R1 much shorter than pterostigma. Legs with tarsal claws simple. **Body measurements (mm).** Body L: 4.9; fore wing L: 4.4; ovipositor sheaths L: 0.85 (approximate measurement); metafemur L/W: 1.10/0.45; metatibia L: 1.54; metatibia inner/outer spurs L: 0.16/0.15; first segment of metatarsus L: 0.50; F1/2/3/14/15/16: 0.30/0.28/0.27/0.15/0.14/0.21; ocular–ocellar line: 0.24; interocellar distance: 0.22; posterior ocellus diameter: 0.10.

##### Distribution.

United States: TX.

##### Etymology.

Named after my brother Jorge Luis, as appreciation for his love and for all the experiences we have lived together over the years (including helping me to collect insects).

##### Comments.

No biological or molecular data is known for this species.

#### Microplitis juanmanueli

Taxon classificationAnimaliaHymenopteraBraconidae

Fernandez-Triana sp. n.

http://zoobank.org/1057486E-01AC-4E02-ABB9-D7D37AEC7D1D

Figs 8

##### Holotype.

Female, CNC, UNITED STATES. Holotype locality: Doolittle Ranch, Mount Evans, 2987m, Colorado, USA.

##### Holotype labels.

First label: Doolittle Ranch/9800’ Mt Evans,/COLO. 3-VIII/S. M. Clark ’61. Second label: CNC497179.

##### Paratypes.

3 ♀, 2♂ (CNC). USA, CO, Echo Lake, Mount Evans, 2,926–3,231m. **Other material examined.** 1 ♀ (CNC). Canada, BC, Atlin. Voucher codes: CNC281008, CNC281009, CNC281011, CNC281019, CNC841840, CNC841841.

**Figures 8.**
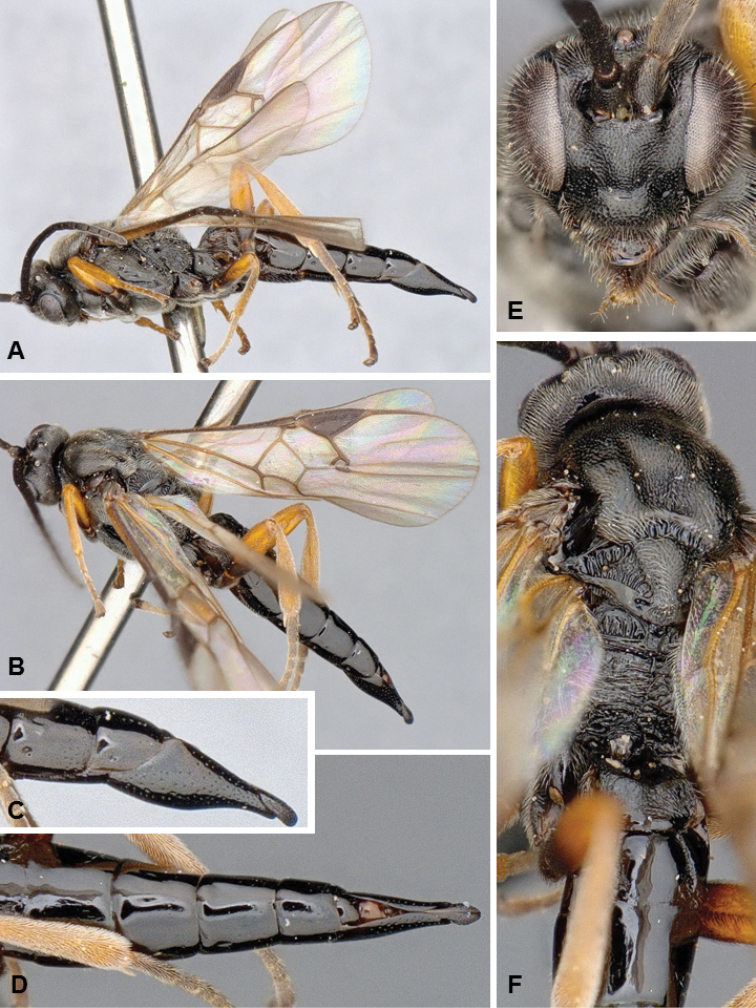
*Microplitis juanmanueli*, female holotype.

##### Diagnosis.

The extremely long metasoma of female (longer than the combined length of head and mesosoma) is unlike any other known *Microplitis*. That character, altogether with the very elongated hypopygium (which is almost twice as long as the last tergite, and considerably projects beyond it), the distinctive shape and sculpture of the ovipositor sheaths, and the elongated mouth parts, allow to unequivocally recognize the species.

##### Description.

Body dark brown to black, legs mostly orange-yellow (except for coxae, anterior 0.1–0.2 of femora and metatarsus which are dark brown to black). Wings hyaline, with most veins dark brown but some veins on anterior half of wings (e.g., M+Cu and 1A) yellowish. Head and mesosoma extensively and coarsely sculptured, metasoma mostly smooth (except for strongly sculptured T1) and with very few setae on tergites. Hypopygium with relatively deep but sparse punctures. Ovipositor sheaths with strong sculpture (striae and punctures) on most of its surface. Head elongate, malar line longer than mandible base; labrum not enlarged; mandibles not enlarged nor strongly curved; glossa long; clypeus and face bulging centrally. Metasoma extremely elongate, longer than combined length of head and mesosoma, and representing approximately 0.6 of entire body length. Hypopygium very elongate, projecting considerably beyond last tergum. Ovipositor sheaths widened and rounded at apex. Fore wing with vein R1 slightly shorter than pterostigma. Legs with tarsal claws pectinate. **Body measurements (mm).** Body L: 5.7 (5.4–6.4); fore wing L: 4.2 (4.2–4.4); metasoma L: 3.6 (3.2–3.9); hypopygium L: 1.00 (0.98–1.06) ovipositor sheaths L: 0.45 (0.42–0.52); metafemur L/W: 1.01/0.30 (0.97–1.01/0.29–0.31); metatibia L: 1.38 (1.28–1.34); metatibia inner/outer spurs L: 0.20/0.20 (0.19–0.20/0.19–0.20); first segment of metatarsus L: 0.45 (0.42–0.43); F1/2/3/14/15/16: 0.23/0.26/0.24/0.13/0.11/0.17 (0.23–0.25/0.25–0.27/0.22–0.24/0.13/0.11–0.12/0.16–0.17); ocular–ocellar line: 0.19 (0.18); interocellar distance: 0.16 (0.16–0.17); posterior ocellus diameter: 0.09 (0.08–0.09).

Male. As in female, but metasoma of normal proportions.

##### Distribution.

Canada, BC; United States, CO.

##### Etymology.

This truly unique and exceptional species is named after my brother Juan Manuel, as appreciation for his love and for all the experiences we have lived together over the years (including helping me to collect insects). Praying and hoping you can defeat the terrible cancer you are battling!

##### Comments.

Because the long metasoma is only found in female specimens (also related to unique shape and sculpture of hypopygium and ovipositor sheaths), it can be argued that those features are somehow related to parasitism; however until host caterpillars are found no further speculation is possible. This is one of the largest, most distinctive and unique species of Microgastrinae: at 6.4 mm of body length, one of the paratypes possibly represents the largest (although not the bulkiest) microgastrine wasp ever collected in North America –and indeed, even in the world very few species surpass that body length. However that size is only attained due to the disproportionately long metasoma (fore wing lengths, at 4.2–4.4 mm, are similar to that of large species of *Microplitis* and many other genera of Microgastrinae; as it is the rest of the wasp body). Beyond length, the species is also notable because of the shape and sculpture of hypopygium and ovipositor sheaths, head (with elongate mouth parts and clypeus and face bulging centrally), and shape and sculpture of T1-T2. In spite of so many unique morphological features, we still consider this species to belong to *Microplitis*, although whenever molecular data becomes available, the generic status might be revisited. The location of the female specimen from Atlin (Canada, BC) is thousands of kilometers apart from the localities of the Colorado specimens, but no morphological differences to separate them could be found. Until more is known, all are kept as one species (although the Canadian specimen is not considered as a paratype). No biological or molecular data is known for this species.

#### Microplitis julioalbertoi

Taxon classificationAnimaliaHymenopteraBraconidae

Fernandez-Triana sp. n.

http://zoobank.org/51630C1F-BC61-4453-AF33-B2E7F15F56E6

Fig. 9

##### Holotype.

Female, CNC, UNITED STATES. Holotype locality: Millen, Georgia, USA.

##### Holotype labels.

First label: Millen Ga./25.VIII.1957/J.G. Chillcott. Second label: CNC666523.

##### Paratype.

1 ♀ (CNC), United States: GA, Hiawassee, Towns County, 610m.

##### Other material.

1 ♀ (CNC), United States: CT, East Hartford.

**Figures 9.**
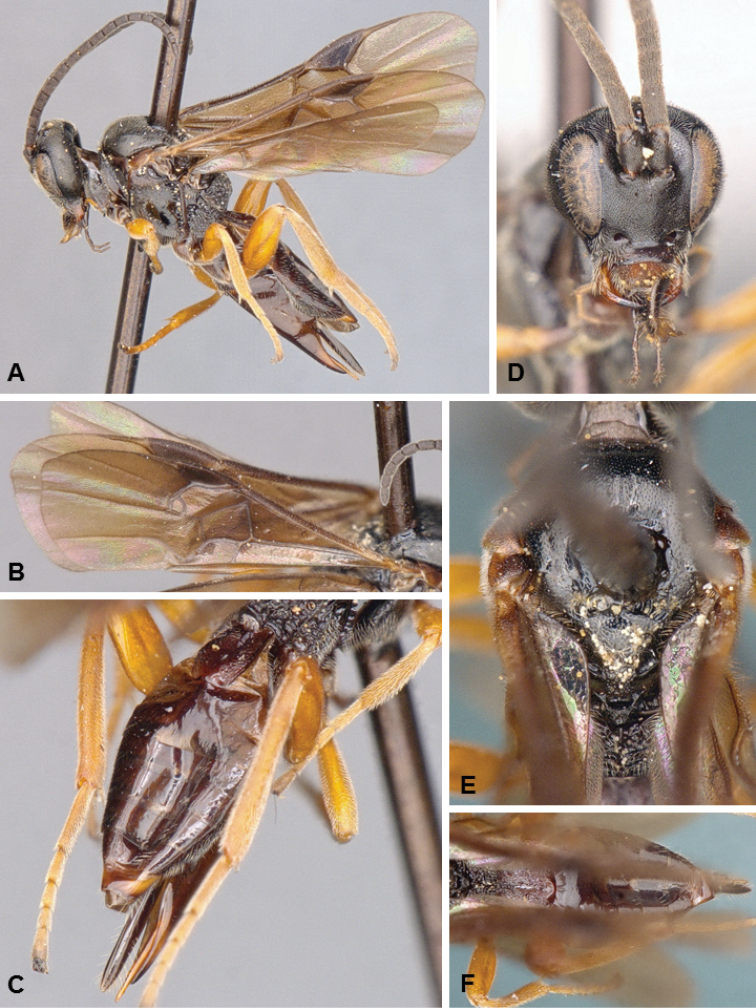
*Microplitis julioalbertoi*, female holotype.

##### Diagnosis.

This is one of three species of *Microplitis* described in this paper with elongate mouth parts (the other two being *M. juanmanueli* and *M. mariamargaritae*). Until now, no *Microplitis* in North America had been reported to have that character. *M. julioalbertoi* has a much shorter and stout metafemur (2.6 × as long as wide; as compared with 2.8–3.3 × in the other two species). It further differs from *M. mariamargaritae* in having an enlarged and acute hypopygium, with much longer ovipositor sheaths; and it can be separate from *M. juanmanueli* because of its normal-sized metasoma (metasoma being extraordinarily large in *M. juanmanueli*). *M. julioalbertoi* is also similar to *M. jorgeluisi*, but that species does not have elongated mouth parts, its palpi are yellow (dark brown in *M. julioalbertoi*) and the ovipositor sheaths do not have any setae (ovipositor with apical 0.3 with numerous setae which are as long as ovipositor sheaths width in *M. julioalbertoi*).

##### Description.

Body reddish-brown, legs mostly orange-yellow (except for coxae). Wings entirely infumated, veins dark brown. Head finely sculptured, mesosoma and metasoma mostly smooth dorsally (except for strongly sculptured T1). Ovipositor sheaths smooth, with setae as long as sheaths width. Head elongate; malar line longer than mandible base; labrum large; mandibles very long and strongly curved; glossa elongate. Hypopygium elongate and sharply acute, projecting considerably beyond last tergum. Ovipositor sheaths relatively long. Fore wing with vein R1 much shorter than pterostigma. Legs with tarsal claws simple. **Body measurements (mm).** Measurements (mm). Body L: 5.2 (5.2–5.5); fore wing L: 4.2 (4.3–4.5); ovipositor sheaths L: 0.95 (0.77–0.83); metafemur L/W: 1.04/0.40 (1.10/0.45); metatibia L: 1.54 (1.64); metatibia inner/outer spurs L: 0.16/0.16 (0.16/0.15); first segment of metatarsus L: 0.48 (0.49); F1/2/3/14/15/16: 0.27/0.26/0.25/0.14/0.13/0.20 (0.28/0.27/0.25/0.15/0.15/0.19); ocular–ocellar line: 0.24 (0.22); interocellar distance: 0.22 (0.17); posterior ocellus diameter: 0.09 (0.09).

##### Distribution.

United States: GA, CT.

##### Etymology.

Named after my brother Julio Alberto, as appreciation for his love and for all the experiences we have lived together over the years (including helping me to collect insects).

##### Comments.

The location of the female specimen from CT is roughly 1,500 kilometers north of the specimens from GA. However, no morphological differences to separate them could be found, and thus all are kept as one species for now (although the CT specimen is not considered as a paratype). No biological or molecular data is known for this species.

#### Microplitis mariamargaritae

Taxon classificationAnimaliaHymenopteraBraconidae

Fernandez-Triana sp. n.

http://zoobank.org/A6D67F7D-36A3-40B3-9249-546C82FCE646

Fig. 10

##### Holotype.

Female, CNC, UNITED STATES. Holotype locality: Great Sand Dunes National Park and Preserve, 2316m, Colorado, USA.

##### Holotype labels.

First label: COLO., Great Sand/Dunes Nat. Mon./1.VIII.68, 7600’/E.C.Becker. Second label: CNC666524.

**Figures 10.**
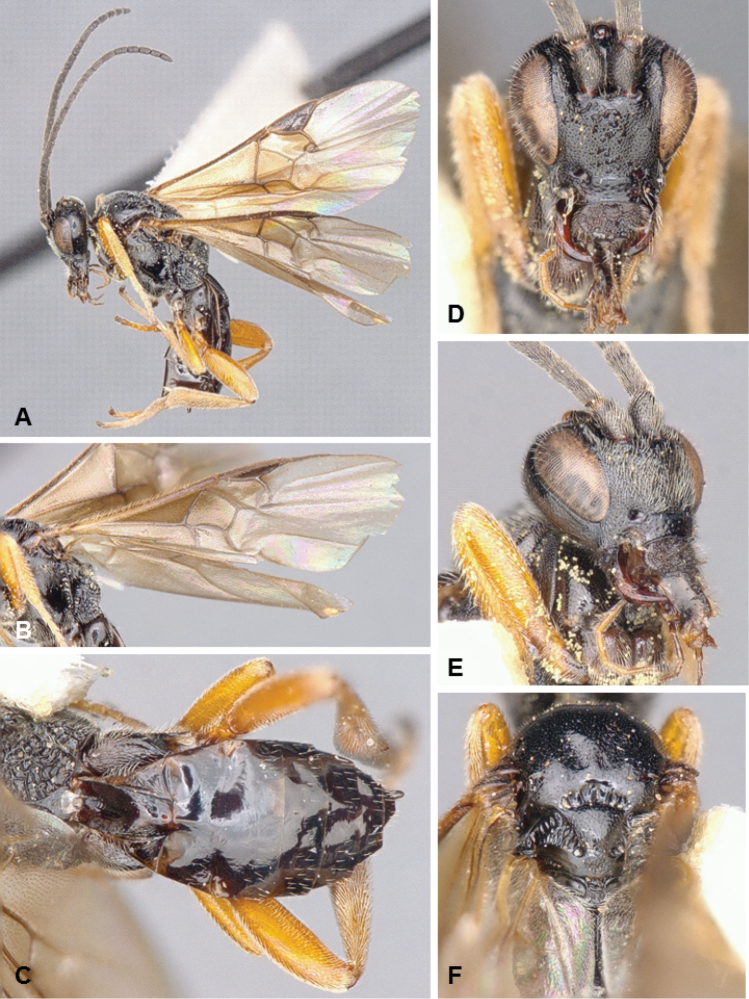
*Microplitis mariamargaritae*, female holotype.

##### Diagnosis.

This is one of three species of *Microplitis* described in this paper with elongate mouth parts (the other two being *M. juanmanueli* and *M. julioalbertoi*). Until now, no *Microplitis* in North America had been reported to have that character. *M. juanmanueli* and *M. julioalbertoi* have enlarged and modified hypopygiums, whereas *M. mariamargaritae* has a normal sized hypopygium (i.e., not extending significantly beyond the tip of metasoma). Also *M. mariamargaritae* has a relatively narrow T1 (3.0 × as long as wide at posterior margin) which is parallel-sided for most of it length and then narrows toward posterior margin (thus anterior margin of tergite is wider than posterior margin); whereas in both *M. juanmanueli* and *M. julioalbertoi* T1 is relatively wider (at most 2.5 × as long as wide at posterior margin), with tergite widening towards posterior margin and only slightly narrowing on posterior 0.2 or less (but even then having anterior and posterior margins of tergite about the same width).

##### Description.

Body dark brown to black, legs mostly orange-yellow (except for coxae). Wings infumated on anterior 0.6, with most veins dark brown. Head coarsely sculptured, mesosoma and metasoma mostly smooth dorsally. Ovipositor sheaths smooth. Head elongate; malar line longer than mandible base; labrum large; mandibles very long and strongly curved; glossa elongate. Fore wing with vein R1 much shorter than pterostigma. Legs with tarsal claws simple. **Body measurements (mm).** Body L: 4.3; fore wing L: 4.2; ovipositor sheaths L: 0.40; metafemur L/W: 1.06/0.38; metatibia L: 1.42; metatibia inner/outer spurs L: 0.15/0.15; first segment of metatarsus L: 0.55; F1/2/3/14/15/16: 0.30/0.28/0.27/0.17/0.15/0.20; ocular–ocellar line: 0.20; interocellar distance: 0.19; posterior ocellus diameter: 0.09.

##### Distribution.

United States: CO.

##### Etymology.

Named after my sister María Margarita, as appreciation for her love and for all the experiences we have lived together over the years (including helping me to collect insects).

##### Comments.

No biological or molecular data is known for this species. Until now, seven endemic insect species had been recorded from the Great Sand Dunes National Park and Preserve (https://www.nps.gov/grsa/learn/nature/insects.htm). Thus *Microplitis mariamargaritae* becomes the eight endemic species from that significant natural area.

**Figure 11.**
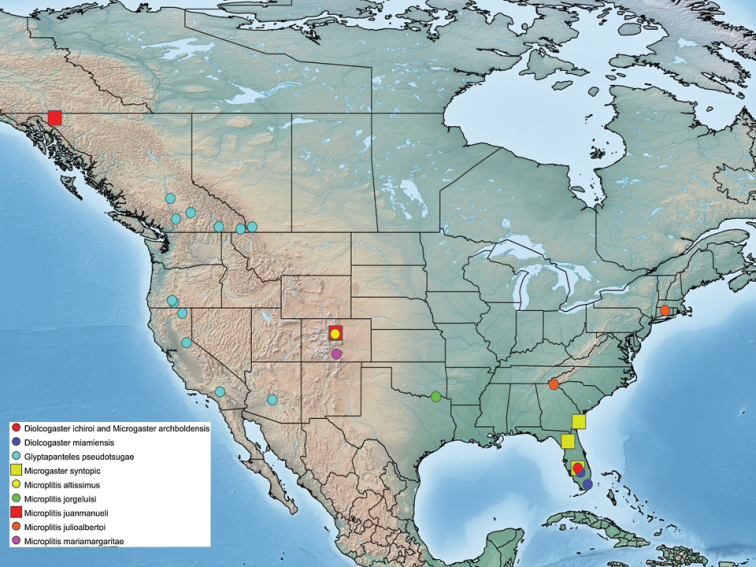
Distribution map of the ten newly described species of Microgastrinae in North America.

## Supplementary Material

XML Treatment for Diolcogaster ichiroi

XML Treatment for Diolcogaster miamensis

XML Treatment for Glyptapanteles pseudotsugae

XML Treatment for Microgaster archboldensis

XML Treatment for Microgaster syntopic

XML Treatment for Microplitis altissimus

XML Treatment for Microplitis jorgeluisi

XML Treatment for Microplitis juanmanueli

XML Treatment for Microplitis julioalbertoi

XML Treatment for Microplitis mariamargaritae
